# Angiotensin Receptor–Neprilysin Inhibitor in Heart Failure Patients With Renal Dysfunction

**DOI:** 10.1155/2024/6231184

**Published:** 2024-11-04

**Authors:** Xiaogang Zhu, Xialing Li, Lingxuan Zhu, Zichuan Tong, Xiuying Xu

**Affiliations:** ^1^Department of Cardiology, Fu Xing Hospital, Capital Medical University, Beijing, China; ^2^School of Data Science, The Chinese University of Hong Kong, Shenzhen, China; ^3^Department of Cardiology, Beijing Tiantan Hospital, Capital Medical University, Beijing, China

**Keywords:** angiotensin receptor–neprilysin inhibitor, chronic kidney disease, heart failure, renal dysfunction, sacubitril/valsartan

## Abstract

Heart failure (HF) and renal dysfunction often coexist and interact in many complex and bidirectional pathways, leading to detrimental effects on patient outcomes. The treatment of HF patients with renal dysfunction presents a significant clinical challenge. Interestingly, sacubitril/valsartan, an angiotensin receptor–neprilysin inhibitor (ARNI), may have beneficial effects on cardiac and renal outcomes in patients with HF with reduced ejection fraction, particularly by slowing the rate of decrease in the estimated glomerular filtration rate compared to a single angiotensin–converting enzyme inhibitor. Recently, more reports have emphasized the renal protection of sacubitril/valsartan in patients with HF. In HF patients with renal dysfunction, however, there is no strong evidence supporting the use of sacubitril/valsartan to reduce the absolute risk of hyperkalemia and worsening renal function; therefore, the administration of ARNI requires a careful balance between the benefits and risks. Furthermore, the lack of evidence-based management highlights the importance of an individualized approach based on published experience and multidisciplinary collaborations, as well as underlines the need for in-depth studies investigating the underlying mechanisms in cardiorenal interactions with a focus on treatments.

## 1. Introduction

Heart failure (HF) and renal dysfunction often coexist and are closely intertwined, sharing common risk factors such as diabetes mellitus or hypertension. They interact in a complex and interdependent way, triggering vicious cycles that can worsen cardiac and renal function, resulting in detrimental effects on patient outcomes [1]. Renal dysfunction not only frequently overlaps with HF but also increases the risk of toxicities associated with pharmacological treatment for HF and weakens the response to diuretics [2], posing significant challenges for guideline-directed medical therapy (GDMT) for HF patients with renal dysfunction. Interestingly, sacubitril/valsartan, an angiotensin receptor–neprilysin inhibitor (ARNI), may have beneficial effects on cardiac and renal outcomes in patients with heart failure with reduced ejection fraction (HFrEF), particularly by slowing the rate of decrease in the estimated glomerular filtration rate (eGFR) compared to a single angiotensin–converting enzyme inhibitor (ACE-I) (enalapril) [3]. Recently, more reports have emphasized the renal protection of sacubitril/valsartan in HF patients [4–8]. However, it is important to note that transient or long-term changes involving a decrease in eGFR and an increase in serum creatinine (SCr) due to decreased glomerular filtration pressure occur generally when sacubitril/valsartan is initiated [9], similar to other renin–angiotensin–aldosterone system inhibitors (RAASi). This suggests that inhibition of neprilysin with sacubitril may not necessarily offset the effects of inhibition of RAAS on renal function. In this context, the efficacy and, importantly, the safety of ARNI in this population need to be clarified and revisited.

This review is aimed at underlining contemporary knowledge associated with the cardiorenal interaction between HF and renal dysfunction, as well as pharmacology and clinical trials of ARNI in HF patients with renal dysfunction, and providing updates for the application strategies of ARNI in this population.

## 2. Epidemiology

The epidemiology of renal dysfunction in HF differs widely depending on the terminology and population studied. Generally, renal dysfunction refers to a reduction in glomerular filtration rate (GFR) resulting from primary or secondary kidney diseases and can present as chronic kidney disease (CKD) or acute kidney injury (AKI). In KDIGO (Kidney Disease: Improving Global Outcomes) guideline, CKD was defined as a persistent GFR < 60 mL/min/1.73 m^2^ (GFR categories G3a-G5) or one marker of kidney damage (eg, a urine albumin-to-creatinine ratio (UACR) ≥ 30 mg/g) for > 3 months [10, 11]. HF patients often suffer from coexisting chronic renal dysfunction, the latter occurring in approximately 40%–50% of HF patients [1]. In a large meta-analysis of patients with HFrEF and heart failure with preserved ejection fraction (HFpEF), up to 55% of all patients with HF had CKD (eGFR < 60 mL/min per 1.73 m^2^) [12]. However, in patients with HFrEF, the prevalence of advanced CKD (CKD stage ≥ G4) was about 10% [11, 12]. On the other hand, the incidence of HF was higher in patients with CKD, and as the severity of chronic renal dysfunction increased, so did the incidence of HF [13]. In the study of Atherosclerosis Risk in Communities (ARIC), the prevalence of HF in people with CKD was 18/1000 person-years, and the prevalence of new-onset HF in patients with CKD was between 17% and 21% [14]. However, the prevalence of HF in patients with end-stage renal disease (ESRD) on dialysis was as high as 44%, and HFrEF represented 50% of these cases [13].

Currently, AKI is defined as an abrupt decrease (≤ 48 h) in kidney function (that is, an absolute increase in SCr of ≥ 0.3 mg/dL (26.4 *μ*mol/L)), a percentage increase in SCr of ≥ 50%, or a decrease in urine output (recorded oliguria of < 0.5 mL/kg/hour for > 6 h) [1, 15]. Approximately 5%–7% of all hospitalized patients experience AKI of varying severity, and 20% of these patients are hospitalized for HF [1]. Furthermore, the study of Acute Decompensated Heart Failure National Registry (ADHERE) showed that approximately 30% of hospitalized patients with acute decompensated HF (ADHF) had AKI or CKD [16].

## 3. Mechanisms of Cardiorenal Interaction Between HF and Renal Dysfunction

Currently, three primary mechanisms are believed to contribute to the development and progression of heart–kidney and kidney–heart interactions: (1) hemodynamic changes resulting from the low cardiac output and increased venous return; (2) dysregulation of the (neuro) hormonal axis through activation of the sympathetic nervous system (SNS) and the natriuretic peptide system, as well as activation of the RAAS; (3) and other factors that exacerbate HF and CKD (e.g., chronic inflammation, diabetes mellitus, and hypertension) [1]. Furthermore, recognition of the bidirectional links between heart and kidney function, as well as understanding that dysfunction of one organ can impact the other, led to the introduction of the term “cardiorenal syndrome” (CRS), which was classified into five subtypes based on disease acuity and sequential organ involvement [17]. Importantly, all three mechanisms are interconnected and contribute to negative effects on both heart and kidney function.

### 3.1. Hemodynamic Mechanisms

HF resulting from cardiac remodeling and congestion can lead to a state of low cardiac output. The traditional explanation for the development of CRS is that the failing heart has a weak ability to generate forward flow, leading to prerenal hypoperfusion. Inappropriate renal afferent flow can activate the (neuro) hormonal axis, increase renal vasoconstriction, and cause renal tubular hypoxia and necrosis, ultimately resulting in loss of renal function [1, 18]. However, the process of “forward failure” only partially explains the hemodynamic mechanisms of CRS, especially the mechanism of renal dysfunction in the acute clinical setting. A case in point is that many hospitalized patients with acute CRS had normal left ventricular ejection fraction (LVEF) and maintained arterial blood pressure [19]. Additionally, the ADHERE registry study showed that the incidence of increased SCr was similar in ADHF patients with reduced and preserved ejection fraction [16], suggesting that HFpEF and HFrEF may share mechanisms that result in renal impairment, despite the greater heterogeneity in the pathophysiology of HFpEF relative to HFrEF [9, 20].

It is interesting to note that despite not being vital organs for transporting oxygenated blood, the kidneys receive a substantial fraction (25%) of cardiac output due to low-resistance circuits [18]. Importantly, renal filtration fraction and plasma flow can be maintained at a steady rate through tubuloglomerular feedback mechanisms, which are independent of renal perfusion pressure within the limits of autoregulation [21]. However, the difference between arterial driving pressure and venous outflow pressure must be significant enough to ensure adequate renal blood flow and glomerular filtration. In this sense, increased central venous pressures (CVPs) can lead to renal venous hypertension, elevated renal resistance, and ultimately impaired intrarenal blood flow in patients with severe decompensated HF, which can be observed by invasive hemodynamic monitoring [22, 23]. Additionally, in patients with persistent renal venous hypertension, both the glomeruli and the tubules are involved, leading to tubular hypertrophy, tubulointerstitial renal fibrosis, and intraglomerular sclerosis. On the contrary, renal venous hypertension can also cause an increase in CVP, as well as salt and water retention resulting in fluid overload. Hence, renal venous congestion (hypertension) may play a critical role in accelerating cardiac and renal dysfunction in patients with CRS [1].

### 3.2. (Neuro) Hormonal Mechanisms

Activation of the SNS and RAAS is considered crucial in HF and renal dysfunction.^1^ Persistent activation of RAAS may be at the core of the pathophysiology of cardiorenal interactions, exerting detrimental effects on both the heart and the kidneys through a series of reactions mediated by increased Angiotensin II and aldosterone [1, 24]. Sodium and water retention, as well as renal and systemic vasoconstriction induced by RAAS activation, can cause increased inflammation and oxidative stress, elevated venous return, and end-diastolic ventricle pressure. Furthermore, activation of RAAS can lead to a rapid increase in renal and cardiac remodeling by promoting fibrosis and increased galectin-3, which eventually results in further decreases in cardiac and renal function (ie, CKD/AKI, and worsening HF). Furthermore, increased SNS activity can further exacerbate RAAS-mediated fluid overload, enhance systemic vasoconstriction, and impair catecholamine clearance due to a reduction in renal function induced by renal vasoconstriction (primarily preglomerular vasoconstriction), which accelerates the overall progression of renal and cardiac dysfunction [18, 25–27].

**Figure 1:**
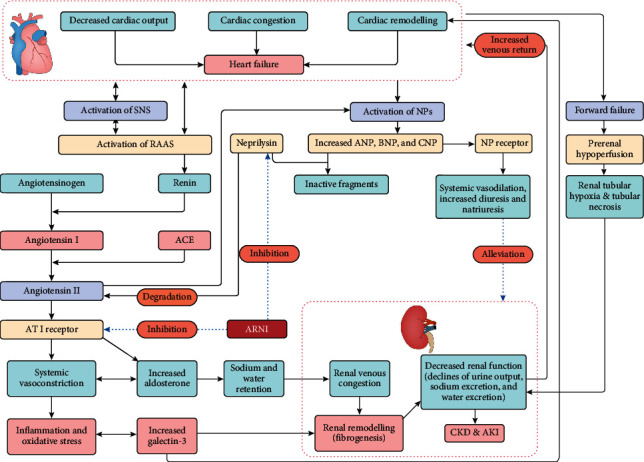
Mechanisms in cardiorenal interactions and points of interest for ARNI. The failing heart has a weak ability to generate forward flow (“forward failure”), leading to prerenal hypoperfusion that can activate the (neuro) hormonal axis and increase renal vasoconstriction, thereby resulting in renal tubular hypoxia and tubular necrosis. Moreover, a state of low cardiac output can result in misregulation of the (neuro)hormonal axis by way of activation of SNS and NPs and triggering of RAAS. Persistent RAAS activation can exert detrimental effects on both the heart and the kidneys via a series of reactions mediated by increased angiotensin II and aldosterone, eventually resulting in further decreased cardiac and renal function. Importantly, ARNI can exert dual angiotensin receptor and neprilysin inhibition. Abbreviations: ACE, angiotensin-converting enzyme; AKI, acute kidney injury; ANP, A-type natriuretic peptide; ARNI, angiotensin receptor-neprilysin inhibitor; AT1 receptor, angiotensin type 1 receptor; BNP, B-type natriuretic peptide; CKD, chronic kidney disease; CNP, C-type natriuretic peptide; NPs, natriuretic peptides; RAAS, renin-angiotensin-aldosterone system; SNS, sympathetic nervous system.

It should be noted that activation of RAAS and SNS leads to elevated cardiac ventricular preload and afterload, increased wall stress due to sodium and water retention and vasoconstriction, and subsequent generation of natriuretic peptides (A-type natriuretic peptide (ANP), B-type natriuretic peptide (BNP) and C-type natriuretic peptide (CNP)), with ANP and BNP being produced in large quantities [28]. Stimulation of NPs may also be directly influenced by the release of Angiotensin II and aldosterone [1, 24]. Although both ANP and BNP promote natriuresis, diuresis, and vasodilation, as well as inhibit RAAS and cardiac fibrosis, they are insufficient to counteract the harmful effects of upregulated RAAS on the dysfunctional heart and kidneys. The resulting imbalance between RAAS and NPs, as well as the reduced renal response to ANP, further suggest that HF is a state of (neuro) hormonal imbalance [1, 29]. In the context of (neuro) hormonal imbalance in HF, it is important to consider the effects of other hormones, such as arginine vasopressin and its precursor, which can further increase the burden on the impaired heart and kidneys (for example, dilutional hyponatremia) [30, 31].  Figure 1 highlights the hemodynamic and (neuro) hormonal mechanisms involved in cardiorenal interactions.

### 3.3. Cardiovascular Disease (CVD)-Associated Mechanisms

CVD and its comorbidities associated with HF and renal dysfunction include arterial hypertension, systemic atherosclerosis, diabetes mellitus, wasting and cachexia, cardiorenal anemia, mineral and bone disorders, and chronic inflammation. These factors are also believed to contribute to the development and progression of HF, AKI, or CKD [1, 32–34].

## 4. Development and Pharmacology of Sacubitril/Valsartan

It is now widely recognized that natriuretic peptides not only serve as reliable markers of HF severity and prognosis [35] but also play a critical role in the management of HF. During the past few decades, two strategies have been employed to modulate the natriuretic peptide pathway in an attempt to improve the outcomes in HFrEF. The first involves the administration of intravenous nesiritide, a recombinant human BNP. Although this exogenous natriuretic peptide initially demonstrated promising effects on natriuresis and hemodynamics in patients with HFrEF [36], it failed to improve outcomes of acute HF in a large-scale randomized controlled trial (RCT) [37]. The second strategy involves inhibiting the degradation of natriuretic peptides. Physiologically, neprilysin can cleave natriuretic peptides and other vasoactive peptides (e.g., bradykinin, Angiotensin II, and Substance P) into inactive fragments [24]. Therefore, inhibiting neprilysin can lead to an increase in the serum level of natriuretic peptides (e.g., BNP) by prolonging its half-life [38], thereby resulting in a variety of actions, including diuresis, natriuresis, vasodilatation, and antagonism of the RAAS [39]. Early studies showed that medications inhibiting neprilysin alone did not improve outcomes in patients with HF [40, 41], possibly because neprilysin also degrades other vasoactive peptides, such as Angiotensin II. Administration of a neprilysin inhibitor alone can raise serum natriuretic peptide and Angiotensin II levels, with the latter activating RAAS and counteracting the beneficial effects of natriuretic peptides [42]. Furthermore, the clinical development of omapatrilat, a medication containing a neprilysin inhibitor in combination with an ACE-I, was terminated due to disappointing results, including an unacceptable incidence of life-threatening angioedema [43, 44]. This adverse effect was attributed to omapatrilat inhibiting three proteases (neprilysin, ACE, and aminopeptidase P) that can break down bradykinin, leading to an unintended excessive potentiation of bradykinin, resulting in vasodilation and increased vascular permeability [44, 45]. As a result, the field's focus on neprilysin in HF faded out for nearly two decades.

Until recently, LCZ696 (sacubitril/valsartan) was developed to inhibit neprilysin while also counteracting the adverse effects of RAAS and reducing excessive bradykinin potentiation. LCZ696 is the first dual-acting crystalline complex composed of the ARB (angiotensin–receptor blocker) valsartan and the neprilysin inhibitor prodrug sacubitril. Shortly after oral ingestion, LCZ696 is broken down into sacubitril and valsartan [24, 45], and sacubitril is then metabolized into an active sacubitrilat (LBQ657) that effectively inhibits neprilysin and increases the beneficial effects of natriuretic peptides. Unlike omapatrilat, sacubitrilat does not inhibit aminopeptidase P, which is expected to reduce the incidence of angioedema [24]. Valsartan can inhibit RAAS activation by blocking the Angiotensin II-1 (AT1) receptor without directly interfering with bradykinin metabolism (see Figure 1). However, in the context of impaired kidneys, valsartan may increase SCr due to decreased intraglomerular pressures resulting from ARB-mediated greater vasodilation in the efferent arteriole than in the afferent arteriole. Furthermore, sacubitril and valsartan can act synergistically, leading to sustained inhibition of neprilysin and RAAS over a 24-h period when administered twice daily. This promotes vasodilation, natriuresis, and diuresis, as well as preventing or reversing the effects of cardiovascular remodeling [24, 46]. Furthermore, in experimental animal models, sacubitril/valsartan may ameliorate glomerulosclerosis, fibrosis, and tubulointerstitial injury and improve renal function compared to valsartan therapy alone [47, 48].

Interest in neprilysin has been revived in recent years with the development of ARNI and the groundbreaking results of PARADIGM-HF (Prospective Comparison of ARNI With ACEI to Determine Impact on Global Mortality and Morbidity in Heart Failure Trial) [49]. In this large and geographically diverse RCT of patients with HFrEF, sacubitril/valsartan significantly reduced the risk of primary endpoint events by 20% compared to enalapril [49]. The neprilysin inhibition component of LCZ696 was believed to be the key to the clinical benefit observed in PARADIGM-HF [50]. Based on the results of this landmark trial, the latest US guidelines have included sacubitril/valsartan as first-line treatment in combination with GDMT for patients with symptomatic HFrEF [51]. However, the US guidelines prohibit ARNI therapy for patients with a history of angioedema due to the concern that it will increase the risk of a recurrence of angioedema [51], although the investigators concluded that LCZ696 did not increase the risk of serious angioedema compared with ACE-I in PARADIGM-HF trial (19/4187 vs. 10/4212, *p* = 0.13) [49]. Furthermore, due to the increased vasodilator effects resulting from dual angiotensin receptors and neprilysin inhibition, treatment with ARNI was associated with a higher incidence of symptomatic hypotension [52]. However, this rarely led to discontinuation of treatment. Most importantly, given the links between HF and kidney disease, as well as the dual pathway inhibition of LCZ696, further investigation into the impact of ARNI is essential in patients with HF, particularly those with kidney dysfunction.

## 5. Evidence for the Effects of ARNI on Renal Function in HF Patients With or Without Renal Dysfunction

Concurrent CKD is an independent predictor of increased mortality and morbidity in HF [9], so medications that improve cardiovascular outcomes and reduce the progression of CKD should be prioritized in HF patients with CKD. Although the efficacy of sacubitril/valsartan in reducing hospitalizations and mortality in HFrEF is well established, data on its effects on renal function in HF patients with renal impairment are limited. Most clinical HF trials that focused on cardiovascular outcomes excluded patients with CKD, so the tolerability and efficacy of ARNI in patients with renal impairment, particularly those with moderate to advanced CKD, remain uncertain. As a result, recommendations for patients with CKD often rely on extrapolation from subgroup analyses or populations with small numbers, without CKD, or anecdotal evidence that has inherent limitations.  Table 1 summarizes pivotal trials associated with the effect of ARNI on composite renal outcomes in patients with HF, Table 2 focuses on the effect of ARNI on glomerular filtration, and Table 3 highlights its effect on serum potassium [3, 4, 49, 53–57].

In the PARADIGM-HF trial that excluded patients with an eGFR < 30 mL/min/1.73 m^2^, there was no significant difference in the rate of composite renal outcome or hyperkalemia between the LCZ696 group and enalapril group (2.2% vs. 2.6%, *p* = 0.28; 16.1% vs. 17.3%, *p* = 0.15, respectively) [49]. In an analysis of the CKD subgroup of PARADIGM-HF with an eGFR of 30–60 mL/min/1.73 m^2^ at screening, sacubitril/valsartan led to a slower rate of decrease in eGFR compared to enalapril (−0.80 vs. −1.55 mL/min/1.73 m^2^ per year, *p* < 0.001), despite causing a modest increase in UACR (1.2 mg/mmol vs. 0.90 mg/mmol, *p* < 0.001) [3]. Increased urinary albumin excretion is generally associated with a faster worsening of renal function, and decreased eGFR and increased UACR are linked to increased mortality and hospitalizations for HF in HFrEF patients with CKD [58, 59]. However, Damman et al. reported that in patients treated with sacubitril/valsartan, the increase in UACR may simply reflect improved renal perfusion due to the improvement in HF status [3]. Such a statement could not be totally true. In fact, the increase in UACR could reflect a hyperfiltration at a glomerular level that is one of the reasons for renal damage if prolonged over time. Notably, in a retrospective study based on the real-world data, Hsu et al. concluded that the worsening renal perfusion index predicted the clinical outcome of HF patients treated with ARNI [60]. In a secondary analysis of the diabetes subgroup of PARADIGM-HF, Packer et al. reported the role of ARNI in attenuating the effect of diabetes on the deterioration of renal function in patients with HFrEF [56]. In the PIONEER-HF trial, the incidences of hyperkalemia and worsening of renal function did not differ significantly between the sacubitril/valsartan group and the enalapril group, although ARNI reduced NT-proBNP (N-terminal pro-BNP) levels in patients with median LVEF of 24% who were hospitalized for ADHF compared to enalapril [54]. In the latest study evaluating the relative treatment effects of ARNI across the KDIGO risk categories in HFrEF patients, Chatur et al. reported the kidney protective benefits and safety of ARNI across the spectrum of baseline kidney risk [61].

In addition, there are several cohort studies on the effects of ARNI on renal function in the real-world population of HF. In a study of 108 patients with HFrEF, eGFR levels improved significantly in patients treated with sacubitril/valsartan compared to those treated with standard HF care without ARNI (73.8 vs. 61.2 mL/min/1.73 m^2^, *p* < 0.001). Moreover, at the end of the follow-up after therapy with sacubitril/valsartan, eGFR levels increased significantly compared to the baseline levels (70.1 vs. 64.8 mL/min/1.73 m^2^, *p* = 0.04) [62]. Similarly, in another study of 54 consecutive outpatients with HFrEF (53.7% had CKD at baseline), renal function improved during a 12-month follow-up period compared to historical controls who received standard HF care (*p* < 0.001) [5]. In a prospective study of 26 patients with HFrEF and advanced CKD, George et al. concluded that ARNI can be administrated with care in HFrEF patients with CKD Stages 4 and 5 under monitoring of eGFR and potassium [63]. In a postmarking study of 5802 older patients (≥ 75 years) with HF, ARNI was associated with a lower incidence of AKI and hyperkalemia compared with ARBs [64].

It is noteworthy, however, that to date, there are no data on ARNI that convincingly reduced total hospitalizations for HF and cardiovascular death among patients with HFpEF or HFmrEF (heart failure with mildly reduced ejection fraction) with CKD, although renal benefits of ARNI have been reported in several studies of HFpEF [4, 53, 55, 57]. Therefore, current guidelines recommend that ARNI treatment can be considered to decrease hospitalizations for HF in selected patients with HFpEF (particularly those with LVEF on the lower end of this spectrum) and to reduce the risk of hospitalization for HF and cardiovascular death in patients with HFmrEF (Class IIb recommendation) [9, 51, 58]. However, finding a balance between the optimization of clinical outcomes in CKD and HF among patients with impaired renal function without reduced ejection fraction still requires validation in large prospective multicenter clinical studies. In the United Kingdom Heart and Renal Protection-III (UK HARP-III) trial, Haynes et al. reported that ARNI was well tolerated and had similar effects on kidney function and albuminuria to irbesartan in CKD patients with an eGFR of 30 to 60 mL/min/1.73 m^2^, while there were few cases of HF in this trial [65]. Furthermore, in post hoc parallel trial analyses of PARADIGM-HF and PARAGON-HF (the prospective comparison of ARNI with ARB global outcomes in HFpEF) [49, 53], continuation of ARNI did not increase the risk of deterioration in kidney function in HF patients who had an eGFR < 30 mL/min/1.73 m^2^ at least once in follow-up compared with renin–angiotensin system inhibitor (*p* > 0.05) [66]. Also, in an analysis of the TRANSITION study [67], most HFrEF patients with concomitant renal dysfunction hospitalized for ADHF tolerated early initiation of ARNI and demonstrated significant improvements in eGFR [68].

Additionally, in a meta-analysis and systematic analysis including six RCTs and eight observational studies, CKD Stages 3–5 patients with HF in the sacubitril/valsartan group had a slower eGFR decline compared with (OR: 0.83, 95% CI: 0.73–0.95, *p* = 0.007) [69]. However, there were two important limitations in this study. First, this meta-analysis involved several observational studies that lacked experimental randomization to accurately evaluate outcomes. Second, RCTs regarding the role of ARNI among patients with eGFR < 30 mL/min/1.73m^2^ were still rare, and further large-scale RCTs need to be performed to validate the above results. In another systematic review and meta-analysis including 17 RCTs (study drug sacubitril/valsartan or omapatrilat), there was no significant difference in chronic renal events (OR 0.92 [0.8–1.05]) or hyperkalemia (OR 1.02 [0.84–1.23]) between the study group and control group [70], while the study duration ranged widely from several weeks to dozens of months, as well as sacubitril/valsartan and the control drug dosage varied from study to study, meaning an unsatisfactory comparability and a relatively low power of evidence. In general, the current evidence suggests that sacubitril/valsartan does not convincingly lead to an absolute reduction in the risk of renal dysfunction and hyperkalemia, although it may result in a slower rate of decrease in eGFR compared to inhibition of the renin-angiotensin system alone. Just as NPs cannot counteract the adverse effects of RAAS on cardiac and renal function, the benefits of inhibiting neprilysin are also insufficient to offset the effect of RAASi on increased SCr and potassium. Therefore, renal protection provided by sacubitril/valsartan in patients with HF with renal dysfunction should be appropriately deemphasized. On the contrary, more attention should be paid to the safety of sacubitril/valsartan in this population, especially in those with advanced CKD.

## 6. Therapeutic Considerations

Management of ANRI in HF patients with renal impairment presents significant challenges for several reasons. The pharmacodynamics and pharmacokinetics of many drugs, including ARNI, are altered in the face of changing renal function [71]. Although it is beyond the scope of this paper to review all considerations of ARNI in HF patients with impaired kidneys, cardiologists and nephrologists must be aware of the possible renal impacts and adjust dosing regimens or avoid nephrotoxic agents (e.g., certain antibiotics and contrast media). In addition, in HF patients with renal dysfunction, there is a substantial knowledge gap due to the lack of high-level data. Most therapeutic recommendations for the use of ARNI in this population are based mainly on observational studies, extrapolation from subgroup analyses, or populations of HF without renal dysfunction, as noted above. Additionally, the evidence base for the efficacy and safety of ARNI in patients with HFpEF or HFmrEF is limited, thus treatment strategies focus mainly on patients with HFrEF, which cannot be generalized to all patients with HF. Furthermore, the implementation of disease-specific pathophysiology–based management strategies associated with ARNI poses unique challenges in daily clinical practice, despite the definition and classification of CRS [17]. Recognizing these limitations, optimizing the use of ARNI in HF patients with renal dysfunction requires an individualized strategy based on available data and multidisciplinary interactions involving specialists in cardiology and nephrology until further data become available.

The main treatment strategies for ARNI in patients with HFrEF/HFmrEF with renal dysfunction include the following applications: (1) avoidance of ARNI in patients with general contraindications, such as a history of angioedema, symptoms of hypotension, or a SBP (systolic blood pressure) < 90 mmHg; (2) consideration of ARNI in patients with ESRD undergoing maintenance dialysis; (3) avoidance of ARNI in patients with severe renal dysfunction or a rapid decline in renal function or moderate to severe hyperkalemia; (4) consideration of ARNI in patients with mild renal dysfunction or hyperkalemia.

### 6.1. General Contraindications to ARNI in Patients With HFrEF

In patients with chronic HFrEF or acute HFrEF, after hemodynamic stability has been achieved, the use of ARNI is recommended to improve symptoms, reduce the risk of HF hospitalization, and increase survival [9, 48, 51, 54]. However, general contraindications to ARNI apply to HF patients with or without renal dysfunction. It is important to note that patients who intend to initiate ARNI should first rule out a history of angioedema, as this increases the life-threatening risk of a recurrence of angioedema. Additionally, ARNI is contraindicated in patients who have received ACE-I treatment within the last 36 h to minimize the risk of angioedema. Other contraindications to ARNI in patients with HFrEF include known allergic reaction or other adverse reaction, known bilateral renal artery stenosis, pregnancy or risk of pregnancy during treatment, breastfeeding, or symptoms of hypotension or SBP < 90 mmHg [9].

### 6.2. ESRD Undergoing Maintenance Dialysis

Small studies and case reports have demonstrated the efficacy and safety of ARNI in HF patients with ESRD on maintenance hemodialysis or peritoneal dialysis [72–74], suggesting that sacubitril/valsartan is well tolerated in those patients when used appropriately. However, in a retrospective study, Lee et al. reported that 17.4% of HFrEF patients with ESRD on dialysis experienced symptomatic hypotension (SBP < 100 mmHg) [75], which commonly occurred during or immediately after hemodialysis. Therefore, in HF patients with ESRD on maintenance dialysis, it seems reasonable to start sacubitril/valsartan with a low dose of 25–50 mg once daily, monitor blood pressure during management, and gradually adjust the dose according to tolerance [76].

### 6.3. Severely Reduced Kidney Function or Moderate to Severe Hyperkalemia

Undoubtedly, the use of ARNI in nondialysis HF patients with advanced renal disease presents unique challenges due to the lack of data on the use of ARNI in this population. A major concern for physicians and patients is the potential for worsening of renal function or significant hyperkalemia. Current guidelines address the use of sacubitril/valsartan in patients with kidney insufficiency and suggest the possibility of renal dysfunction and hyperkalemia with this medication, particularly in those with comorbid CKD ≥ G4 [9, 11]. Hence, for HF patients with an eGFR lowering to < 30 mL/min/1.73 m^2^, an Scr > 266 umol/L (3 mg/dL), an increase in Scr of > 50% above baseline after ARNI therapy, or moderate to severe hyperkalemia defined as serum potassium > 5.5 mmol/L [9, 76], ARNI use should be contraindicated or discontinued, as should concomitant nephrotoxic drugs and other K^+^ supplements or retention agents (e.g., triamterene and amiloride). In particular, nonsteroidal anti-inflammatory drugs (NSAIDs) should also be avoided unless essential [1, 9]. Furthermore, for those with moderate to severe hyperkalemia, new potassium binders (e.g., patiromer or sodium zirconium cyclosilicate) can help mitigate RAASi-related hyperkalemia, particularly in vulnerable populations of HF with CKD [77]. However, these treatments also require consideration of possible side events, such as gastrointestinal upset, hypomagnesemia, and binding of concurrent oral medications [78].

### 6.4. Mildly Reduced Kidney Function or Mild Hyperkalemia

Regarding the use of ARNI, although mildly reduced kidney function (eGFR 30–60 mL/min/1.73 m^2^) or mild hyperkalemia (serum potassium 5.0–5.5 mmol/L) may be considered acceptable [9], the potential for renal insufficiency and hyperkalemia with this medication still exists. However, fears of inducing hyperkalemia or worsening renal function may contribute to the underuse of this lifesaving therapy in patients with HFrEF. Consequently, for HFrEF patients with mildly reduced kidney function or mild hyperkalemia, it is reasonable to initiate sacubitril/valsartan with a reduced dose (24/26 mg twice daily), double the dose at intervals of ≥ 2 weeks while monitoring tolerability, recheck renal function and electrolytes (BUN, creatinine, K^+^) 1–2 weeks after starting and 1–2 weeks after final dose titration. The monitoring should continue until the patient is stable and at regular intervals afterwards. Additionally, stopping concomitant nephrotoxic drugs and other K^+^ supplements or retaining agents and reducing the dose of diuretics when appropriate should also be considered [9]. It is important to note that an interdisciplinary approach may be necessary, including the early involvement of cardiologists and nephrologists who have a great deal of experience and knowledge of complex patients and are used to facing comorbidities, due to the complexity of interactions between the heart and kidneys [1, 65]. Closer collaboration between cardiologists and nephrologists has been advocated as a mechanism to promote clinical research on HF with dysfunction [79, 80], thus resulting in better long-term persistence of this life-prolonging drug (i.e., ARNI) and better management of recurrent worsening renal function or hyperkalemia attacks.

**Figure 2:**
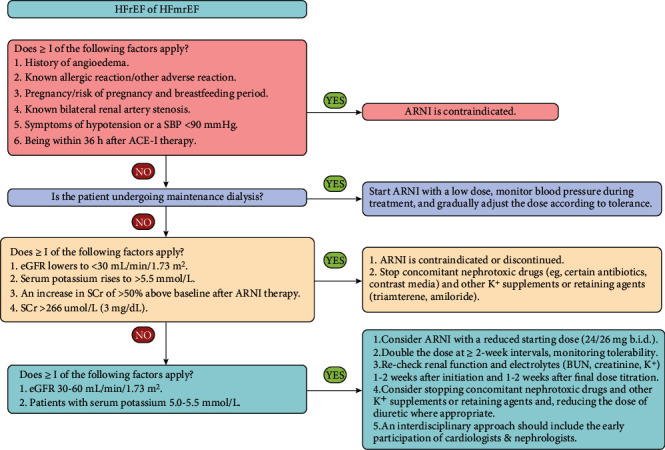
Algorithm for ARNI application in patients with HFrEF/HFmrEF with renal dysfunction. Abbreviations: ACE-I, angiotensin-converting enzyme inhibitor; b.i.d., bis in die (twice daily); BUN, blood urea nitrogen; eGFR, estimated glomerular filtration rate; HFmrEF, heart failure with mildly reduced ejection fraction; HFrEF, heart failure with reduced ejection fraction; SBP, systolic blood pressure; SCr, serum creatinine.

Finally, based on reasoning from the first principles, current guidelines, available evidence, and our experience, we propose an algorithm for ARNI application in patients with HFrEF/HFmrEF with kidney dysfunction **(**Figure 2) and suggest that the management team apply local expertise to fill in the uncertainties.

## 7. Conclusions and Future Directions

Administrating ARNI in HF patients with renal dysfunction presents a significant clinical challenge, and as the incidence of these disorders increases, this challenge will become even more apparent in the future. From a pathophysiological perspective, the heart and kidneys share many interrelated pathways, including altered hemodynamics and fluid overload, as well as significant imbalances in hormonal and sympathoadrenergic status. Dual angiotensin receptor and neprilysin inhibition with sacubitril/valsartan represent a novel approach to the treatment of patients with HFrEF. However, strong evidence supporting the use of sacubitril/valsartan to reduce the absolute risk of hyperkalemia and worsening renal function in HF patients with renal dysfunction is still lacking, although it can cause a slower rate of decline in renal function compared to renin–angiotensin system inhibition alone. There are also concerns about the safety of sacubitril/valsartan among HF patients with renal dysfunction, particularly in advanced CKD. The application of sacubitril/valsartan in the setting of renal dysfunction requires a careful balance between benefits and risks. Furthermore, the lack of evidence-based management highlights the importance of an individualized approach based on published experience and multidisciplinary collaborations, as well as underlines the need for in-depth studies investigating the underlying mechanisms in cardiorenal interactions in HF patients with renal dysfunction with a focus on treatments, especially implementing larger prospective, long-term follow-up studies, including RCTs.

## Figures and Tables

**Table 1:** Effect of sacubitril/valsartan on composite renal outcome in patients with HF.

**Study**	**Comparator**	**Patient population, ARNI/comparator (** **n** **)**	**Median duration of follow-up**	**Composite renal outcome, n (%)**		**p** ** value**
				**ARNI**	**Comparator**	
PARADIGM-HF, McMurray et al. [49]	Enalapril	Patients with HFrEF, 4187/4212	27 months	94 (2.2)	108 (2.6)	0.28
PARAMOUNT, Voors et al. [4]	Valsartan	Patients with HFpEF, 149/152	36 weeks	7 (6)	16 (13)	0.08
PARADIGM-HF (analysis of the CKD subgroup), Damman et al. [3]	Enalapril	HFrEF Patients with CKD, 1333/1412	48 months	22 (1.7)	36 (2.6)	> 0.05
PARAGON-HF, Solomon et al. [53]	Valsartan	Patients with HFpEF, 2407/2389	35 months	33 (1.4)	64 (2.7)	< 0.05
PIONEER-HF, Velazquez et al. [54]	Enalapril	HFrEF Patients hospitalized for ADHF, 440/441	8 weeks	60 (13.6)	65 (14.7)	> 0.05
PARAGON-HF (prespecified analysis), Mc Causland et al. [55]	Valsartan	Patients with HFpEF, 2407/2389	48 months	33 (1.4)	64 (2.7)	< 0.05

*Note:* Composite renal outcome was defined as a decrease in the estimated glomerular filtration rate (eGFR) of > 30–< 60 mL/min/1.73 m^2^ and a decrease of 25% or more in the eGFR from the baseline value or end-stage renal disease (ESRD).

Abbreviations: ADHF, acute decompensated heart failure; ARNI, angiotensin receptor-neprilysin inhibitor; CKD, chronic kidney disease; HF, heart failure; HFpEF, heart failure with preserved ejection fraction; HFrEF, heart failure with reduced ejection fraction.

**Table 2:** Effect of sacubitril/valsartan on glomerular filtration in patients with HF.

**Study**	**Comparator**	**Patient population, ARNI/comparator **(**n**)	**Median duration of follow-up**	**Mean change from baseline in eGFR (mL/min per 1.73 m** ^**2**^ ** per year)**		**p** ** value**
				**ARNI**	**Comparator**	
PARAMOUNT, Voors et al. [4]	Valsartan	Patients with HFpEF, 149/152	36 weeks	−2.2^a^	−7.5^a^	NR
PARADIGM-HF (analysis of the CKD subgroup), Damman et al. [3]	Enalapril	HFrEF Patients with CKD, 1333/1412	48 months	−0.80	−1.55	< 0.001
PARADIGM-HF (analysis of the diabetes subgroup), Packer et al. [56]	Enalapril	HFrEF Patients with type 2 diabetes, 1907/1877	44 months	−1.70	−2.30	< 0.0001
PARAGON-HF (Prespecified analysis), Mc Causland et al. [55]	Valsartan	Patients with HFpEF, 2407/2389	48 months	−2.0	−2.7	< 0.001

Abbreviations: eGFR, estimated glomerular filtration rate; NR, not reported.

^a^Calculated.

**Table 3:** Effect of sacubitril/valsartan on serum potassium in patients with HF.

**Study**	**Comparator**	**Patient population, ARNI/comparator (** **n** **)**	**Median duration of follow-up**	**Incidence of moderate to severe hyperkalemia**		**p** ** value**
				**ARNI**	**Comparator**	
PARAMOUNT, Solomon et al. [57]	Valsartan	Patients with HFpEF, 149/152	36 weeks	24 (16%)	16 (11%)	0.21
PARADIGM-HF, McMurray et al. [49]	Enalapril	Patients with HFrEF, 4187/4212	27 months	674 (16.1)	727 (17.3)	0.15
PARAGON-HF, Solomon et al. [53]	Valsartan	Patients with HFpEF, 2407/2389	35 months	316 (13.1)	361/2367 (15.1)	0.048
PIONEER-HF, Velazquez et al. [54]	Enalapril	HFrEF patients hospitalized for ADHF, 440/441	8 weeks	51 (11.6)	41 (9.3)	> 0.05

*Note:* Moderate to severe hyperkalemia was defined as serum potassium > 5.5 mmol/L.

## Data Availability

The data used to support the findings of this study are available from the corresponding author upon request.
